# Students’ age and parental level of education influence COVID-19 vaccination hesitancy

**DOI:** 10.1007/s00431-021-04343-1

**Published:** 2021-12-22

**Authors:** Anna Zychlinsky Scharff, Mira Paulsen, Paula Schaefer, Fatma Tanisik, Rizky Indrameikha Sugianto, Nils Stanislawski, Holger Blume, Bernhard M. W. Schmidt, Stefanie Heiden, Meike Stiesch, Anette Melk

**Affiliations:** 1 Department of Pediatric Hematology and Oncology, Hannover Medical School, Hannover, Germany; 2 Department of Pediatric Kidney, Liver, and Metabolic Diseases, Hannover Medical School, Hannover, Germany; 3 Department of Prosthetic Dentistry and Biomedical Material Research, Hannover Medical School, Hannover, Germany; 4 Institute of Microelectronic Systems, Leibniz University Hanover, Hannover, Germany; 5 Department of Nephrology and Hypertension, Hannover Medical School, Hannover, Germany; 6 Institute of Innovation Research, Technology Management & Entrepreneurship, Leibniz University Hanover, Hannover, Germany

**Keywords:** Vaccine hesitancy, COVID-19, Adolescents, Children, Vaccination

## Abstract

Widespread vaccination in pursuit of herd immunity has been recognized as the most promising approach to ending the global pandemic of coronavirus disease 19 (COVID-19). The vaccination of children and adolescents has been extensively debated and the first COVID-19 vaccine is now approved in European countries for children aged > 12 years of age. Our study investigates vaccination hesitancy in a cohort of German secondary school students. We assessed 903 students between age 9 and 20 in the period between 17 May 2021 and 30 June 2021. 68.3% (*n* = 617) reported intention to undergo COVID-19 vaccination, while 7% (*n* = 62) did not want to receive the vaccine and 15% (*n* = 135) were not yet certain. Age and parental level of education influenced COVID-19 vaccine hesitancy. Children under the age of 16 as well as students whose parents had lower education levels showed significantly higher vaccine hesitancy.

*  Conclusion*: Identifying subsets with higher vaccination hesitancy is important for targeting public information campaigns in support of immunization.

**Table:** 

**What is Known:** *• The willingness to receive COVID-19 vaccination among adults in Europe is about 70%, but data for children and adolescents is lacking.* *• The lack of immunization in younger cohorts represents a significant barrier to achieving herd immunity, and also leaves children and adolescents vulnerable to acute and long-term morbidity from natural COVID-19 infections.*	
**What is New:** *• Intention-to-vaccinate among children and adolescents is high (~ 70%); conversely, vaccination hesitancy is low.* *• Age and parental level of education influenced COVID-19 vaccine hesitancy among children and adolescents.*

**Supplementary information:**

The online version contains supplementary material available at 10.1007/s00431-021-04343-1.

## Introduction

Since the development of novel vaccines against coronavirus disease 19 (COVID-19), widespread vaccination in pursuit of herd immunity has become the most promising path to end the pandemic [1]. Most COVID-19 vaccines are approved exclusively for adults. The BNT162b2 COVID-19 vaccine, produced by BioNTech/Pfizer, was initially approved for individuals over the age of 16 and this was expanded to those over 12 years old in June 2021, though the recommendations by the German vaccine regulating agency were more restrictive [2]. The lack of immunization in younger cohorts represents a significant barrier to achieving herd immunity, and leaves children and adolescents vulnerable to acute and long-term morbidity from natural COVID-19 infections [3]. While children represent a minority of severe disease courses and deaths in comparison to adults, they carry a significant burden of disease [4]. Children with underlying conditions and infants under 1 year of age are at risk for severe initial infection [4], and children of all ages, can develop multi-system inflammatory disease in children (MIS-C, also known as PIMS-TS), even after asymptomatic or pauci-symptomatic COVID-19 [5].

As trials demonstrating the safety and efficacy of COVID-19 vaccines in pediatric populations emerge, policy makers worldwide are embarking on campaigns to vaccinate their youngest citizens, making the question of vaccine hesitancy in this group more relevant than ever. Germany, like other European governments, announced on 11 May 2021 that it intends to offer vaccinations to 12- to 17-year olds by the end of the summer school break. Studies examining COVID-19 vaccination intention and hesitancy in this context have focused on parental views, as parents and guardians generally make medical decisions for minors under their care [6, 7]. However, the independent views of adolescents themselves have not been assessed, and this represents a significant informational void in the quest to vaccinate and protect pediatric populations.

In this study, we show that older children, those for whom the vaccine was approved from the initial launch, have higher rates of intention-to-vaccinate than younger ones. In addition, we observed that a lower parental educational level correlated to higher vaccine hesitancy. Conversely, children and adolescents whose parents attained higher education levels were more likely to report that they intended to get the COVID-19 vaccine.

## Methods

### Study design

Data was collected at the final time point of the longitudinal Transmission Analytic COVID-19 (TRAC-19) study, which explored SARS-CoV-2 infections and behavioral patterns in two secondary schools in Hannover, Germany [8]. Nine hundred thirteen students participated in the TRAC-19 study between May 17 and June 30, 2021, and 903 returned the questionnaires about vaccination hesitancy (Suppl. Table 1). In the context of the larger study, students provided nasal swap and blood sample, which were tested for SARS-CoV-2-specific antibodies by Elecsys® Anti-SARS-CoV-2 (Roche) assay according to the manufacturer’s instruction. Venipuncture was completely optional; students could opt-out from blood sampling and still participate in the study.

The TRAC-19 study was approved by the institutional review board (No. 9085_BO_S_2020) and complies with the Declaration of Helsinki. Study participation was voluntary and informed consent was obtained from participants and, in case of minors, their legal guardian.

### Statistics

Data are given as mean and standard deviation (SD) or numbers (*n*) and percentages (%). A multivariable logistic regression model was employed for vaccination hesitancy and was adjusted for multiple comparison with Šidák. For the analysis, two categories were built (yes vs. no/unsure). Covariates included general demographics (sex, age), chronic diseases, and parent’s educational level (one, two, or no adults with college education). Age groups were built according to the categories defined by federal vaccine recommendations: 9–12 years, 13–15 years, and 16–19 years. *P*-values < 0.05 were considered significant. Statistical analyses were performed using SAS 9.4 (SAS Institute Inc., USA).

## Results

A total of 903 students from grades 5 to 13 participated in this study. The mean age was 14.6 years (SD 2.3), and 52.4% were female (Suppl. Table 2). Fifteen percent (*n* = 134) reported having a chronic disease, most commonly asthma, allergies, or attention deficit hyperactivity disorder. Twenty-seven percent (*n* = 246) had no college-educated adult living in their household, 29.8% (*n* = 269) had one, and 28.8% (*n* = 260) had two college-educated adults. Fourteen percent (*n* = 130) of students did not provide sufficient information to determine parental education level and were therefore not categorized in any of the three groups.

**Fig. 1:**
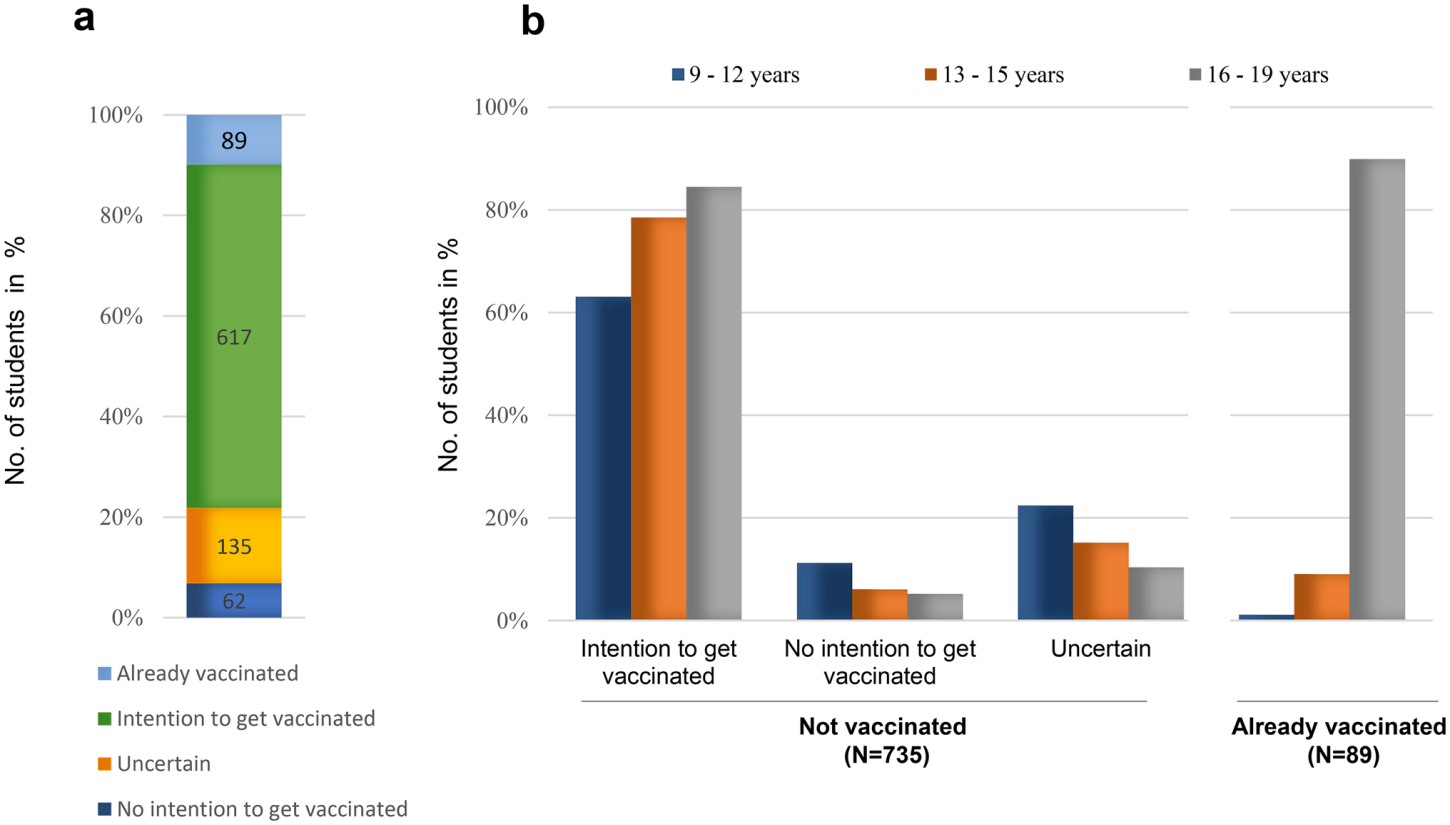
COVID-19 vaccination hesitancy. The intention to undergo COVID-19 vaccination for all students **a** and by age groups for those not vaccinated yet and students already vaccinated (**b**)

Three percent of students (*n* = 28) reported having had COVID-19 (confirmed by polymerase chain reaction or antigen test). We detected SARS-CoV-2 antibodies in 17 of these students and in another 11 students that had not reported a previous positive test. Ten percent (*n* = 89) of students had already received at least one vaccine dose with the majority (*n* = 80) being older than 16 years and thus eligible for the vaccine from the initial release (Fig. 1a). Nine students had received the vaccine despite being below the age of eligibility at the time. Twenty-six of these early vaccinees reported having a chronic disease, accounting for 19.1% (26/136) of all students with chronic diseases.

A total of 903 students (99%) answered the questionnaire about vaccination hesitancy. In addition to those already vaccinated, 68.3% (*n* = 617) reported their intent to undergo COVID-19 vaccination (Fig. 1a). Seven percent (*n* = 62) did not want to receive the vaccine and 15% (*n* = 135) were still uncertain.

We performed a mixed model analysis to identify factors that influence students’ intention to receive the vaccine (Table 1). We observed no sex difference but found differences between the age groups. Older students (age 13–19) showed significantly higher intention-to-vaccinate compared to the younger age group (Fig. 1b, Table 1). Notably, most of the students already vaccinated were adolescents (age 16–19). Students who declined vaccination or were uncertain were likely to belong to the younger age groups (Fig. 1b). To explore the influence of parental education on students’ vaccine preferences, we included the educational status of adults living in the same household, presumably parents or guardians, in the model. Vaccination hesitancy was higher in households with no college-educated adults than in those with one or two college-educated adults (Table 1, Suppl. Table 3). We did not find a significant difference in students’ intention-to-vaccinate between healthy individuals and those reporting chronic condition.

**Table 1:** Logistic regression model for intention-to-vaccinate

Covariate	OR	95% CI		*p*-value
**Sex**				
* Male vs. female*	1.07	0.74	1.55	0.72
**Age**				
* 9–12 vs. 13–15 years*	0.46	0.28	0.75	*0.0005*
* 9–12 vs. 16–19 years*	0.29	0.15	0.54	< *0.0001*
* 13–15 vs. 16–19 years*	0.63	0.33	1.19	*0.22*
**Chronic disease**				
* No vs. yes*	0.51	0.23	1.14	0.10
**College-educated adults**				
* No vs. one*	0.42	0.25	0.72	*0.0003*
* No vs. two*	0.26	0.15	0.46	< *0.0001*
* One vs. two*	0.61	0.33	1.11	0.13

*OR*, odds ratio; *95% CI*, confidence interval

## Discussion

Widespread vaccination is indispensable to ending the pandemic spread of COVID-19 [1]. As data on children and adolescents is lacking, we assessed vaccination willingness or hesitancy among a cohort of 903 students in May and June 2021, shortly after vaccine access was expanded to include adolescents. Overall, the intention-to-vaccinate was high in our cohort of students, mirroring data from adults in Europe showing similar vaccine acceptance [9].

The vaccination of children and adolescents has been hotly debated, especially in the context of in-person learning. While the European Medicines Agency has already approved the BNT162b2 vaccine for children over 12 years of age in May 28, the Standing Committee on Vaccination (STIKO) in Germany has offered more restrictive guidance, recommending the vaccine in this age group only for those at high risk for severe COVID-19 [2], and finally extending the recommendation for all people aged 12 years or above in August 19. This discrepancy likely contributed to the age difference in vaccination hesitancy we observed, as access to vaccination may have affected willingness. Those students whose age placed them squarely in the eligible range according to the initial approval for BNT162b2 were more likely to report an intention to receive the vaccine. Indeed, nearly a third of students in this category had already received the first dose. Students in the 12–16 age group, for whom a vaccination was recommended by the STIKO only in the case of chronic illness during the study period, had higher rates of vaccine hesitancy, suggesting that timing of recommendations and the discrepancy between European and national guidelines may have influenced this cohort. Even though the STIKO vaccination guidelines changed only for children aged 12–16 years with a chronic condition during the observation period, this remains an important limitation to this study. Vaccination hesitancy is often highest in the initial phase of vaccination campaigns and may diminish as time goes on, and widespread vaccination contributes to an aura of normalcy around the newly introduced substance [10].

In our cohort, intention-to-vaccinate was higher in children and adolescents from households with college-educated caregivers. This is consistent with data showing that an educational level below a bachelor’s degree predicted hesitancy towards routine childhood vaccinations and annual influenza vaccines [11]. Brandstetter et al. suggest that parental educational level influences parents’ intention-to-vaccinate their children against COVID-19. However, this study only surveyed parents of younger children (1 to 5 years of age) examining a context in which health care choices are presumably predicated on parental preferences alone, and thus did not address the views of minor patients themselves [12]. We sought to examine an age group that, while still under the legal custody of parents or guardians, is likely to participate in decision-making around their own medical care and whose viewpoints and intentions are thus highly relevant. It is important to note, however, that German law, like in most European countries, requires parental consent for vaccination in individuals under the age of 18 years. While this limits the agency of minors to some extent, a German court ruled that minors may receive a COVID-19 vaccination even against the wishes of one parent, provided the vaccination is in accordance with STIKO guidelines and the minor and the other parent agrees to vaccination [13]. It is interesting to note in the context of Brandstetter et al. [12] that parental education level seems to affect not only parents’ attitude towards COVID-19 vaccines, but also that of their offspring.

Importantly, 15% of our cohort was still uncertain about receiving a COVID-19 vaccine. This hesitant but likely convincible subset is the most relevant target group of public information campaigns in support of immunization. While our study did not explore the reasons underlying the reported uncertainty, previous studies suggest that vaccination safety, concerns over side effects, and believing others in greater need of the vaccine may play a role [7, 14].

## Conclusion

Overall, we report high levels of vaccine willingness, although younger age and lower parental education levels correlated with higher vaccine hesitancy. Identifying subsets with a higher vaccination hesitancy is important for the targeting of public information campaigns in support of immunization and achieving herd immunity.

## Supplementary Information

Below is the link to the electronic supplementary material.

Supplementary file1 (PDF 290 KB)

## Data Availability

De-identified patient datasets will be available upon written request to the corresponding author following publication.

## Abbreviations

COVID-19: Coronavirus disease 19
SARS-CoV-2: Severe acute respiratory syndrome coronavirus 2
SD: Standard deviation
TRAC-19: Transmission Analytic COVID-19
