# Supplementary material for: Students’ age and parental level of education influence COVID-19 vaccination hesitancy
Source: Eur J Pediatr. 2021 Dec 22;181(4):1757–62. doi: 10.1007/s00431-021-04343-1 (PMC8691963; doi:10.1007/s00431-021-04343-1)
Supplement: Supplementary file 1 — Supplementary file1 (PDF 290 KB) [file 431_2021_4343_MOESM1_ESM.pdf]

SUPPLEMENTAL INFORMATION

Students' Age and parental level of education influence COVID-19 vaccination hesitancy

Anna Zychlinsky Scharff*[1], Mira Paulsen*[2], Paula Schaefer[3], Fatma Tanisik[3], Rizky Indrameikha Sugianto[2], Nils Stanislawski[4], Holger Blume[4], Bernhard M.W. Schmidt[5], Stefanie Heiden[6], Meike Stiesch[3], Anette Melk[2]

*Authors contributed equally and are both considered first authors

SUPPLEMENTAL TABLE 1 **Content of additional questions TRAC-19 study – Final time point May/June 2021**

The TRAC-19 study collected data by questionnaires at various time points. The demographic data (age, sex, parental education) was collected at study inclusion. When vaccines became available an additional questionnaire was distributed to all study participants in May/June 2021 asking the following further details:

- If the students has received one, two or no COVID-19 vaccination
- In case the student received vaccination: the date(s) of vaccination and the type of vaccine
- In case the student had not received vaccination, the student was asked whether he would like to receive the vaccine in future with the possible answers yes/no/unsure

SUPPLEMENTAL TABLE 2 **Demographics of study participants**

**Study participation**		**Age (years)**	**Female sex**	**Height (cm)**	**Weight (kg)**	**BMI**	**Chronic disease**	**Medication**	**Household size**	**Parental college education**		
										**No adult**	**One adult**	**Two adults**
	N (%)	Mean (SD)	N (%)	Mean (SD)	Mean (SD)	Mean (SD)	N (%)	N (%)	Mean (SD)	N (%)	N (%)	N (%)
**All students**	**903**	14.6 (2.3)	473 (52.4)	162.9 (13.0)	53.2 (15.5)	20.1 (12.6)	134 (14.8)	144 (15.9)	4.1 (1.1)	246 (27.2)	269 (29.8)	260 (28.8)
9-12 years	260 (28.7)	11.9 (0.7)	128 (49.2)	150.2 (8.0)	40.6 (9.5)	17.8 (3.0)	27 (10.4)	22 (8.5)	4.2 (1.0)	64 (24.6)	74 (28.5)	81 (31.2)
13-15 years	370 (40.9)	14.5 (0.9)	193 (52.2)	164.4 (9.0)	53.2 (12.6)	19.6 (3.7)	65 (17.6)	47 (12.7)	4.2 (1.2)	105 (28.4)	107 (28.9)	107 (28.9)
16-19 years	273 (30.2)	17.5 (0.9)	152 (55.7)	172.3 (12.1)	64.6 (14.6)	22.8 (21.9)	42 (15.4)	75 (27.5)	4.0 (1.2)	77 (28.2)	88 (32.2)	72 (26.4)

SUPPLEMENTAL TABLE 3 **Sensitivity analysis intention-to-vaccinate logistic regression model**

	**Model 1 – excluding missing data sets**			**Model 2 – including missing data sets**		
Covariate	Estimate	SE	p - value	Estimate	SE	p - value
**Intercept**	3.10	0.51	<.0001	3.08	0.47	<.0001
**Sex**						
male	0.07	0.19	0.72	0.18	0.17	0.30
female		Ref.			Ref.	
**Age**						
9 - 12 years	-1.25	0.27	<.0001	-1.22	0.25	<.0001
13 -15 years	-0.47	0.27	0.08	-0.48	0.24	0.05
16 -19 years		Ref.			Ref.	
**Chronic disease**						
no	-0.67	0.41	0.10	-0.66	0.39	0.09
yes		Ref.			Ref.	
**College-educated adults**						
Unclassified/missing	-	-	-	-0.79	0.28	0.0057
none	-1.36	0.24	<.0001	-1.34	0.24	<.0001
one	-0.50	0.25	0.05	-0.49	0.25	0.05
two		Ref.			Ref.	

SE, standard error
